# Abdominal Unicentric Castleman Disease: A Hepato-Pancreatico-Biliary Frenemy

**DOI:** 10.7759/cureus.88762

**Published:** 2025-07-25

**Authors:** Evangelia Florou, Emeema Govindu, Yoh Zen, Parthi Srinivasan, Andreas Prachalias

**Affiliations:** 1 Hepato-Pancreato-Biliary Surgery, King's College Hospital, London, GBR; 2 Pathology and Laboratory Medicine, King's College Hospital, London, GBR; 3 Hepato-Pancreato-Biliary Surgery and Liver Transplantation, London Bridge Hospital, London, GBR

**Keywords:** abdominal mass, castleman disease, hepatopancreatobiliary surgery fdg pet, lymphoproliferative disorder, metabolically active mass, paracaval mass, paraduodenal mass, retroperitoneal mass, retroperitoneal tumour, unicentric castleman disease

## Abstract

Castleman disease (CD) is a group of rare lymphoproliferative disorders characterized by shared histopathological features but distinct clinical entities, broadly classified into unicentric Castleman disease (UCD) and multicentric Castleman disease (MCD). UCD involves a single anatomical site and typically follows a benign clinical course, whereas MCD affects multiple lymph node stations and is associated with systemic symptoms and a more complex therapeutic approach. The disease is poorly understood, and the difficulty in reaching a diagnosis is well noted in the literature. While MCD is systemic and requires hematological work-up, abdominal UCD consists of a radiologically detected solitary mass that poses a diagnostic challenge, often necessitating a hepato-pancreatico-biliary (HPB) opinion.

We report a retrospective case series of four patients diagnosed with UCD between 2011 and 2022 at a tertiary centre. All patients underwent extensive diagnostic work-up due to suspected malignancy based on radiological features and metabolic imaging. Surgical resection was performed in all cases, given diagnostic ambiguity or concern for malignancy.

The cohort included three males and one female, aged 24 to 69 years. Lesions were located in the retroperitoneum, pancreaticoduodenal groove, small bowel mesentery, and adjacent to the caudate lobe. In one patient, UCD coexisted with a head of pancreas adenocarcinoma. In all cases, definitive diagnosis was established following surgical resection and histopathological analysis. One patient was found to have a coexisting focus of follicular dendritic cell sarcoma and remains free of recurrence 12 years post-resection. This rare association has been reported in the context of hyaline-vascular UCD and carries potential malignant behaviour, underscoring the need for long-term surveillance. All patients were referred to hematology services.

UCD carries a low malignant potential; however, affected individuals may have an increased risk of developing lymphoproliferative disorders. HPB surgeons should maintain a high index of suspicion for this rare entity when evaluating retroperitoneal, paraduodenal, or mesenteric masses. In the majority of cases, surgical resection represents the culmination of an often complex diagnostic process that poses significant challenges to clinicians and leads patients to undergo surgery in the absence of a definitive preoperative diagnosis. Although complete surgical resection is considered curative and is typically associated with favourable outcomes, the future role of surgery may be subject to re-evaluation, particularly as advancements in radiological modalities could potentially facilitate non-invasive diagnosis. Regardless of the surgical outcome, all patients should be referred to hematology services for long-term follow-up. This case series underscores the diagnostic difficulties posed by UCD and highlights the importance of multidisciplinary collaboration in the management of such cases.

## Introduction

Castleman disease (CD) consists of a group of haematological disorders that share similar histopathological features, but the aetiologies, clinical presentation, treatment, and outcomes differ widely. The disease was first described by Benjamin Castleman in 1954 in a patient with mediastinal localized lymph node enlargement, with histologically profound lymphoid follicles and regressed germinal centres. Further investigations established the disease by the mid-1980s and classified it into unicentric Castleman disease (UCD), which involves a single site of the body, as opposed to multicentric Castleman disease (MCD), which affects multiple regions of lymph node enlargement [1,2].

Studies have shown an association of MCD with human herpesvirus 8 (HHV-8), which is the aetiological driver in HIV-positive patients. Furthermore, two additional categories have been identified: HHV-8 positive HIV-negative MCD, and idiopathic MCD (iMCD), which is HHV-8 negative [2,3].

UCD typically presents as an incidentally identified solitary mass on imaging studies of the neck, mediastinum, abdominal cavity, retroperitoneum, or pelvis. Most patients are asymptomatic; however, mild compressive symptoms may occur at times [3,4]. Although CD has low malignant potential, it is associated with an increased risk of lymphomas [5].

Undoubtedly, the evaluation of a mass located adjacent to the liver, duodenum, pancreas, inferior vena cava (IVC), or mesentery typically falls under the remit of the Hepato-Pancreato-Biliary (HPB) team. UCD represents a potential diagnostic pitfall in HPB surgery, particularly given its rarity, with fewer than 60 abdominal cases reported in the literature to date [6].

In this case series, we present our experience with the diagnostic work-up and surgical management of this rare pathology. HPB surgeons should be familiar with UCD, including its potential to mimic malignancy, the implications of surgical resection, and considerations for long-term follow-up.

## Case presentation

We conducted a retrospective case series analysis of patients diagnosed with UCD at King’s College Hospital between 2011 and 2022. The objective was to describe the clinical presentation, diagnostic work-up, radiological characteristics, operative management, and outcomes of patients presenting with abdominal or retroperitoneal UCD managed by the HPB surgical team. Institutional records were reviewed to identify patients with histologically confirmed UCD during the study period. Data collected included demographics, presenting symptoms, imaging findings, laboratory investigations, operative details, histopathological subtype, and postoperative follow-up. The study was conducted in accordance with institutional ethical standards, and all patient data were anonymised.

Case 1

**Figure 1:**
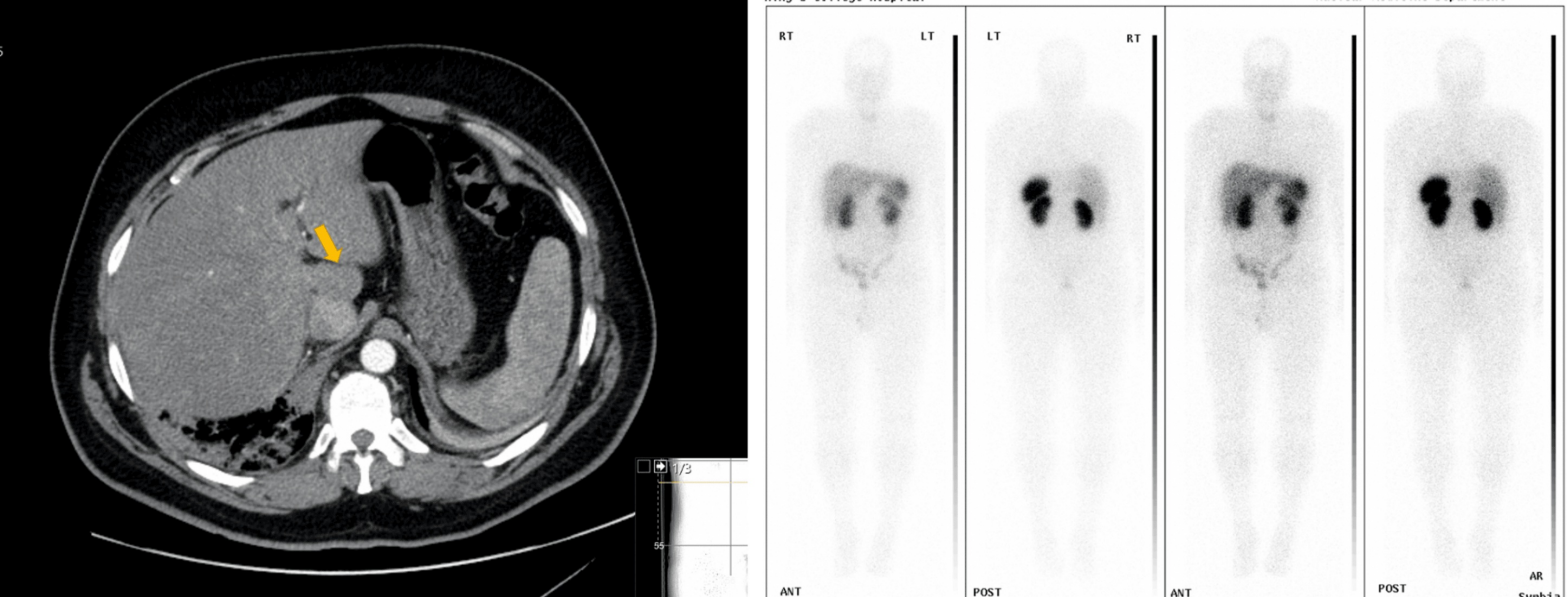
Abdominal mass on work-up, proven to represent UCD. On the left, CT scan showing a mass adjacent to the caudate lobe. On the right, MiBG scan showing no activity at the lesion. This case was treated in 2011, before the advent of DPET, which was introduced into clinical practice in 2016. MiBG: Meta-iodobenzyl-guanidine; DPET: DOTATATE positron emission tomography; UCD: Unicentric Castleman disease.

A 57-year-old male presented with a three-week history of fever and right upper quadrant pain. He was diagnosed with right lower lobe pneumonia, and during his admission, he was found to have deranged liver function tests. The latter prompted further investigations with imaging studies. An ultrasound of the abdomen detected a 5 cm hypoechoic mass adjacent to the IVC, and CT confirmed the presence of a mass adjacent to the caudate lobe in close relationship to the IVC. This was an incidental finding, and the differential diagnosis included neuroendocrine tumour (NET) and paraganglioma. The work-up involved urine catecholamines, tumour markers, virology, NET screen, meta-iodobenzyl-guanidine (MiBG) scan, and octreotide scan (Figure 1). This case was treated in 2011, before the advent of DOTATATE positron emission tomography (DPET) imaging, which was introduced into clinical practice in 2016.

All investigations returned negative results, and the tumour demonstrated no metabolic activity on nuclear medicine imaging. Following discussion at the multidisciplinary team (MDT) meeting, surgical resection was recommended. The mass was excised along with the caudate lobe, and the procedure was uneventful.

Histological examination confirmed the mass as UCD, with a concurrent finding of a 5 mm focus of follicular dendritic cell sarcoma. The patient was referred to haematology services and continues to undergo surveillance for the last fourteen years with no evidence of recurrence.

Case 2

**Figure 2:**
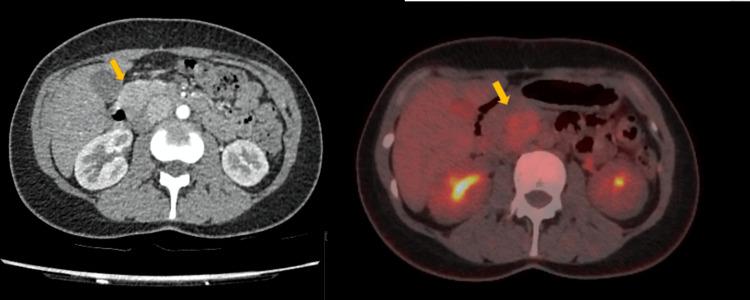
Abdominal mass on work-up, proven to represent UCD. On the left, a CT scan demonstrates a mass (yellow arrow) located within the pancreatico-duodenal groove. On the right, an FDG PET scan shows intense metabolic activity in the mass (yellow arrow). UCD: Unicentric Castleman disease; FDG PET: Fluorodeoxyglucose positron emission tomography.

A 69-year-old female presented with anaemia and a positive faecal occult blood test, raising suspicion for gastrointestinal malignancy. CT revealed a 4 cm mass located within the pancreatico-duodenal groove. The differential diagnosis included a gastrointestinal stromal tumour (GIST) and NET. Biochemical work-up for a functioning NET was negative, and the imaging features were atypical for NET. Due to the mass’s location, tissue sampling was not feasible. A DPET scan demonstrated metabolic activity in the lesion and also identified a second focus of uptake within the pancreatic head. Given these findings, lymphoma was added to the differential diagnosis, prompting further evaluation with fluorodeoxyglucose positron emission tomography (FDG PET). MRI of the pancreas was contraindicated due to a metallic implant, so a repeat CT with a dedicated pancreatic protocol was performed instead. FDG PET confirmed avid uptake in the 4 cm mass, while the pancreatic head exhibited physiological tracer uptake. Consequently, the second DPET-positive area was deemed non-pathological (Figure 2). As the lesion remained uncharacterised and malignancy could not be excluded, a decision was made to proceed with surgical resection.

Intraoperatively, the tumour was found to tether the antimesenteric wall of the duodenum at the end of the second part, and partial duodenal wall resection was required (sleeve duodenectomy). The procedure was uncomplicated, and the patient was discharged a week later. Histological analysis confirmed UCD.

Case 3

**Figure 3:**
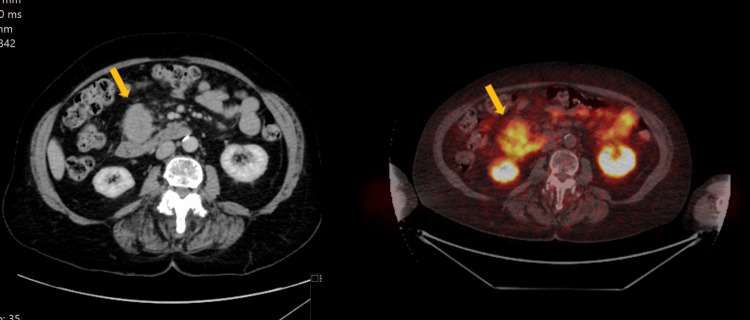
Abdominal mass on work-up, proven to represent UCD. On the left, a CT scan demonstrates a mass (yellow arrow) lying adjacent to the pancreatic head, causing a pressure effect on the adjacent renal vasculature. On the right, an FDG PET scan shows avidity of the mass (yellow arrow). UCD: Unicentric Castleman disease; FDG PET: Fluorodeoxyglucose positron emission tomography.

A 24-year-old female presented with lower back ache. This was a recurrent episode of urinary tract infections, from which she had been suffering for the last four years. A renal ultrasound showed an incidental finding of a mass lying in the aortocaval recess. Given the location of the mass, the case was referred to HPB services for further work-up and management. CT revealed a 3 cm aortocaval mass in close proximity to the head of the pancreas and duodenum, with pressure effect on the adjacent renal vessels. The differential diagnosis included NET, GIST, paraganglioma, and leiomyoma/leiomyosarcoma. The mass was metabolically avid on FDG PET. On MRI, the mass showed restricted diffusion and arterial enhancement with washout (Figure 3).

A biopsy was performed via endoscopic ultrasound (EUS) to aid in diagnosis. The EUS showed a solid, hypoechoic, well-circumscribed mass with a central scar, measuring 3 cm, lying adjacent to the IVC. However, the sample obtained was suboptimal for histological assessment. A repeat EUS-guided biopsy yielded tissue that histologically showed reactive lymphoid hyperplasia. Given the uncertainty of the diagnosis in a young patient with a metabolically avid retroperitoneal mass, the unanimous decision was to proceed with surgical resection.

The para-caval mass was resected alone, as it was not involving adjacent organs, and the procedure was uncomplicated. Complete microscopic analysis of the mass confirmed UCD. Haematology referral was made for long-term follow-up, and the patient remains free of disease seven years post-resection.

Case 4

A 63-year-old male presented with painless obstructive jaundice. Further investigations confirmed a histological diagnosis of adenocarcinoma of the head of the pancreas. A staging CT scan revealed an additional solitary soft tissue mass measuring 5 cm within the small bowel mesentery, raising suspicion for metastatic disease.

**Figure 4:**
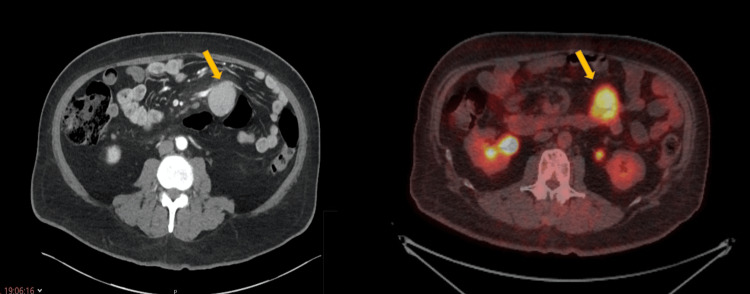
Abdominal mass on work-up, proven to represent UCD. This case had a concurrent biopsy-proven diagnosis of pancreatic cancer. On the left, a CT scan demonstrates a mass (yellow arrow) lying within the pancreatico-duodenal groove. On the right, an FDG PET scan shows intense activity of the mass (yellow arrow), while the primary mass in the head of the pancreas showed no avidity. UCD: Unicentric Castleman disease; FDG PET: Fluorodeoxyglucose positron emission tomography.

To further characterise both lesions and complete staging, an FDG PET scan was performed. Interestingly, the primary pancreatic tumour was not FDG-avid, rendering the study non-diagnostic for both the primary lesion and potential metastatic spread. However, the mesenteric mass demonstrated intense metabolic activity, suggesting a distinct pathological process (Figure 4).

Following MDT discussion, the patient underwent a pancreaticoduodenectomy along with resection of the mesenteric mass. Final histopathological examination of the mesenteric lesion confirmed UCD.

Table 1 summarises the four cases of UCD managed by HPB surgeons.

**Table 1:** Summary of four cases of abdominal UCD managed by the HPB surgery team. Each case includes presenting symptoms, diagnostic work-up, surgical intervention, and final histopathological diagnosis. All patients underwent resection due to diagnostic uncertainty, with UCD confirmed postoperatively. One case (Case 1) demonstrated a rare concurrent finding of follicular dendritic cell sarcoma. UCD: Unicentric Castleman disease; HPB: Hepato-pancreato-biliary surgery;  FDG PET: Fluorodeoxyglucose positron emission tomography; EUS: Endoscopic ultrasound; MIBG: Meta-iodobenzylguanidine; NET: Neuroendocrine tumor.

Case	Presenting Symptoms	Work-up List	Surgical Resection	Final Diagnosis
Case 1	Incidental finding during pneumonia admission	Ultrasound, CT, urine catecholamines, tumor markers, virology, NET screen, MIBG scan, octreotide scan	Yes	UCD + 5 mm follicular dendritic cell sarcoma
Case 2	Anemia, positive fecal occult blood test	CT, biochemical NET screen, DPET, FDG PET, pancreatic protocol CT	Yes	UCD
Case 3	Incidental finding during work-up for lower back pain, recurrent urinary tract infections	Renal ultrasound, CT, FDG PET, MRI, EUS-guided biopsy (twice)	Yes	UCD
Case 4	Painless obstructive jaundice (concurrent with pancreatic adenocarcinoma)	Staging CT, FDG PET	Yes	UCD

Histological challenges

**Figure 5:**
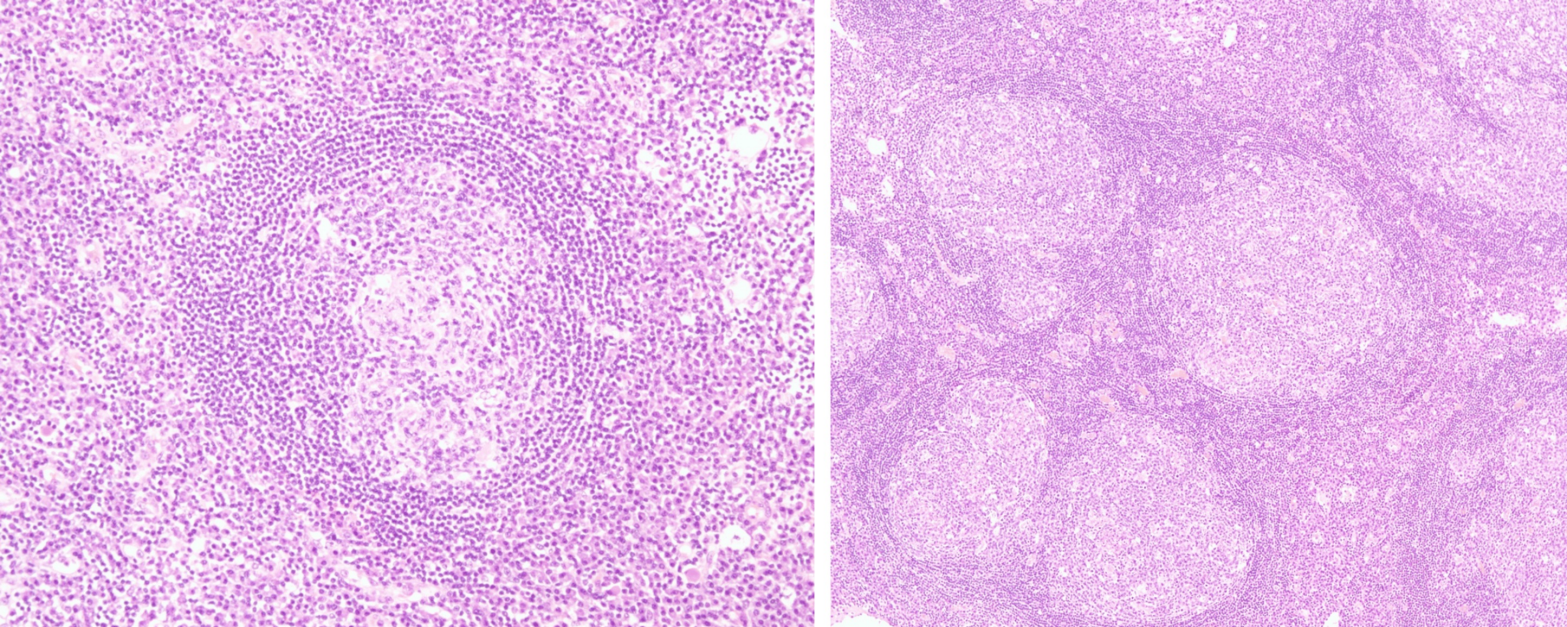
Microscopic examination of UCD. On the left, the lymphoid follicle shows a regressed germinal center and an expanded mantle zone with a concentric cellular arrangement. On the right, some lymphoid follicles exhibit two enlarged germinal centers. UCD: Unicentric Castleman disease.

All cases showed reactive lymphoid hyperplasia with follicular hyperplasia and expansion of interfollicular areas. The follicles were associated with regressed germinal centres, hyalinising arteries, and an expanded mantle zone-findings consistent with UCD. Some follicles also contained multiple enlarged germinal centres (Figure 5). UCD was originally termed ‘giant lymph node hyperplasia’ to describe a benign abnormal growth of lymph nodes. CD has three histological subtypes: the hyaline vascular (HV) type, the plasma cell type, and the mixed type. This classification scheme is moderately correlated with clinical classification (either unicentric or multicentric). Most cases of UCD show HV type histological features, while MCD typically demonstrates plasma cell type morphology. In our series, all four cases were of the HV type, which is the most common, accounting for 70-80% of cases in the literature [3,4]. The vascular hyperplasia within the lymph node mass accounts for its enhancement on arterial phase contrast imaging. Although several characteristic findings of UCD are recognised, they are not specific to this entity. This nonspecificity presents a major preoperative diagnostic challenge, as abdominal UCD can closely mimic other hypervascular or lymphoid lesions such as GIST, paraganglioma, lymphoma, and even metastatic disease [7,8].

## Discussion

The epidemiology of CD remains incompletely understood. MCD is more frequently observed in males, whereas UCD appears to have no clear sex predilection [2-4]. MCD may or may not be associated with HHV-8 and HIV, and thus requires evaluation by haematology teams to guide appropriate management. In contrast, no established risk factors have been identified for UCD, which typically presents in the third to fourth decade of life [2,4]. The estimated incidence of UCD is approximately 21-25 cases per million person-years [9].

CD has been further characterised over the years, with increasing understanding of the radiological, histopathological, and clinical features of UCD. Solitary abdominal UCD accounts for a minority of cases, with retrospective series reporting roughly 11-15% located in the abdominopelvic or retroperitoneal regions, with mediastinal and cervical sites being more common [6,10,11].

Abdominal UCD is most often detected incidentally, with the mass typically asymptomatic or associated with mild compressive symptoms. Anatomical anomalies, such as congenital splenic and renal variations, may coexist and contribute to diagnostic confusion in retroperitoneal or abdominal masses, as highlighted in rare autopsy-based findings [12]. In contrast, mediastinal and cervical UCD can present with more pronounced symptoms. These may include B symptoms, paraneoplastic pemphigus, skin rash, dyspnoea, bronchiolitis, polyneuropathy, and haemolytic anaemia [3].

The radiological features of UCD on CT and MRI are nonspecific. However, cross-sectional imaging plays a crucial role in excluding aggressive malignancies elsewhere in the body and may raise suspicion for UCD [11]. CT is particularly valuable for evaluating the lesion’s anatomical relationship to adjacent organs and major vascular structures [11]. FDG-PET commonly shows focal uptake in abdominal UCD, helping distinguish it from other lesions; however, its findings alone are not diagnostic [10,13,14]. This observation is confirmed in this case series. FDG PET consistently demonstrated metabolic activity within the lesions, which emerged as a key factor in the decision-making process for proceeding with radical surgical resection. The first case described here was treated 13 years ago, and neither biopsy nor FDG PET were performed; a consensus decision for resection was made. In the second case, the proximity of the metabolically active tumour to the pancreas was enough to decide on surgical resection and allow complete histological analysis.

Preoperative histological sampling may be helpful but is often insufficient to establish a definitive diagnosis [15]. In the third case of this series, two attempts at biopsy were made, both of which were inconclusive. In such scenarios, particularly in a young patient with an FDG-PET-avid retroperitoneal mass, the differential diagnosis remains broad, with retroperitoneal sarcoma being a significant concern. Consequently, surgical resection becomes imperative.

Furthermore, biopsy attempts in anatomically challenging locations carry procedural risks and frequently yield inconclusive results, halting diagnostic progress. In such scenarios, surgical exploration may be warranted even in the absence of preoperative histological confirmation. This is particularly relevant in young patients with FDG-avid, radiologically suspicious, and potentially unresectable abdominal masses, where the differential includes UCD and soft tissue sarcomas. In the absence of reliable imaging criteria to distinguish between these entities, both diagnostic and therapeutic uncertainties arise.

In the final case, pancreatic cancer was the primary diagnosis and required definitive treatment. Notably, the pancreatic tumour was not metabolically active on FDG PET, whereas the mesenteric mass, later confirmed to be UCD, demonstrated intense FDG uptake. Had the metabolic pattern been reversed, with only the Castleman lesion exhibiting avidity, the case could have been misinterpreted as metastatic disease. This misclassification could have misled clinicians and potentially denied the patient curative treatment for pancreatic cancer.

In 2016, the Castleman Disease Collaborative Network (CDCN), an international consortium of experts in haematology, haematopathology, haematologic oncology, infectious diseases, and surgery, convened to develop clinical practice guidelines for the management of MCD [16]. In 2020, consensus guidelines for UCD were also published, recommending surgical resection as the treatment of choice [16].

In one large single‑centre series, 30% of UCD cases involved the abdomen or pelvis, and complete resection achieved excellent disease‑free survival [8]. A systematic review of 404 CD cases confirmed that surgery is the definitive treatment for UCD, with 94% undergoing resective procedures and five‑year disease‑free survival exceeding 80%, compared to approximately 34% in multicentric disease [17].

Minimally invasive techniques, including laparoscopic or laparoscopic‑assisted resections, have been successfully applied to mesenteric UCD and hepatic‑hilum lesions, offering reduced postoperative morbidity and shorter hospital stays [15,18].

It is important to note that UCD may be deemed unresectable when involving complex anatomical regions such as the mediastinum or cervical areas. In such cases, neoadjuvant chemotherapy may be considered to reduce tumour size and facilitate future surgical resection [11,13]. For highly vascular lesions, preoperative embolization has also been reported as an adjunct to minimise intraoperative bleeding and improve surgical outcomes [4,9].

When a mass is identified in the upper abdomen in proximity to critical structures such as the liver, duodenum, pancreas, or IVC, referral to HPB specialists is warranted, given the broad differential diagnosis and surgical expertise required for management. Complete surgical excision remains the definitive treatment for UCD, offering excellent long-term outcomes when histologically confirmed [19].

## Conclusions

UCD is now recognized as a distinct entity within the spectrum of hematological disorders. It should be considered in the differential diagnosis of solitary abdominal masses, particularly in asymptomatic patients with incidental findings on imaging. Referral to high-volume centers is recommended for appropriate diagnostic work-up and multidisciplinary management. HPB surgeons may encounter this rare pathology in their practice and should remain vigilant to the diagnostic challenges it poses.

Although UCD has low malignant potential, surgical resection is generally warranted to achieve a definitive diagnosis and curative outcome. All histologically confirmed cases should be referred to hematology services for long-term surveillance. Ultimately, UCD often leads to an extensive diagnostic process, culminating in surgery for a condition with a favorable prognosis, underscoring the importance of awareness and multidisciplinary collaboration in its management.
